# A Puzzle of Vestibular Physiology in a Meniere's Disease Acute Attack

**DOI:** 10.1155/2015/460757

**Published:** 2015-06-18

**Authors:** Marta Martinez-Lopez, Raquel Manrique-Huarte, Nicolas Perez-Fernandez

**Affiliations:** Department of Otorhinolaryngology, Clinica Universidad de Navarra, University of Navarra, Avenida Pío XII 36, 31008 Pamplona, Spain

## Abstract

The aim of this paper is to present for the first time the functional evaluation of each of the vestibular receptors in the six semicircular canals in a patient diagnosed with Meniere's disease during an acute attack. A 54-year-old lady was diagnosed with left Meniere's disease who during her regular clinic review suffers an acute attack of vertigo, with fullness and an increase of tinnitus in her left ear. Spontaneous nystagmus and the results in the video head-impulse test (vHIT) are shown before, during, and after the attack. Nystagmus was initially left beating and a few minutes later an upbeat component was added. No skew deviation was observed. A decrease in the gain of the vestibuloocular reflex (VOR) and the presence of overt saccades were observed when the stimuli were in the plane of the left superior semicircular canal. At the end of the crisis nystagmus decreased and vestibuloocular reflex returned to almost normal. A review of the different possibilities to explain these findings points to a hypothetical utricular damage.

## 1. Introduction

Meniere's disease (MD) crises are usually managed by patients at home or at a primary care facility. The characteristic features of the disease occur and usually hearing loss is best defined and sometimes audiometry is performed to confirm the deterioration in hearing loss.

Case reports of vestibular signs are infrequent because of the limited availability of equipment at emergency rooms. All of them provide interesting insight into the pathophysiology of the disorder by describing findings in terms of spontaneous and post-head-shake or vibration nystagmus, caloric test [1], and more recently head-impulse test (HIT) at bedside or assisted with video (vHIT) for the assessment of semicircular canal function [2] and vestibular evoked myogenic potentials (VEMP) for otolithic function [3].

We present a patient in whom an acute spell of vertigo has been seen while being at the hospital and in which the spontaneous nystagmus and the assessment of all 6 semicircular canals were possible.

## 2. Case Report

A 54-year-old woman was diagnosed with MD in her left ear. Vertigo spells begun 30 years ago, but after three years of activity the disease entered in a quiescent phase. In March 2013 vertigo recurred and since then has been unresponsive to medical treatment. When seen for the first time in July 2014, the number of typical vertigo spells in the 6 months previous was 12; also 4 additional ones were of the Tumarkin type, and her functional level score [4] was 4. PTA in the right ear was 5 dBHL and in her left ear 28 dBHL. CT scan and MRI were normal. After complete evaluation she was treated with intratympanic gentamicin ITG: August 5th and 11th and September 24th. It is interesting to mention that the external auditory canal is markedly anfractuous and the approach to the tympanic membrane is difficult; as such myringotomy was done in the anterior part of the inferior hemotympanum and a curved needle was used to deliver the gentamicin close to the round window niche. The treatment provided 1 month without any symptom and good equilibrium without increased hearing loss. Since then, vertigo spells have recurred scaling up in intensity and again one Tumarkin spell has occurred.

**Figure 1:**
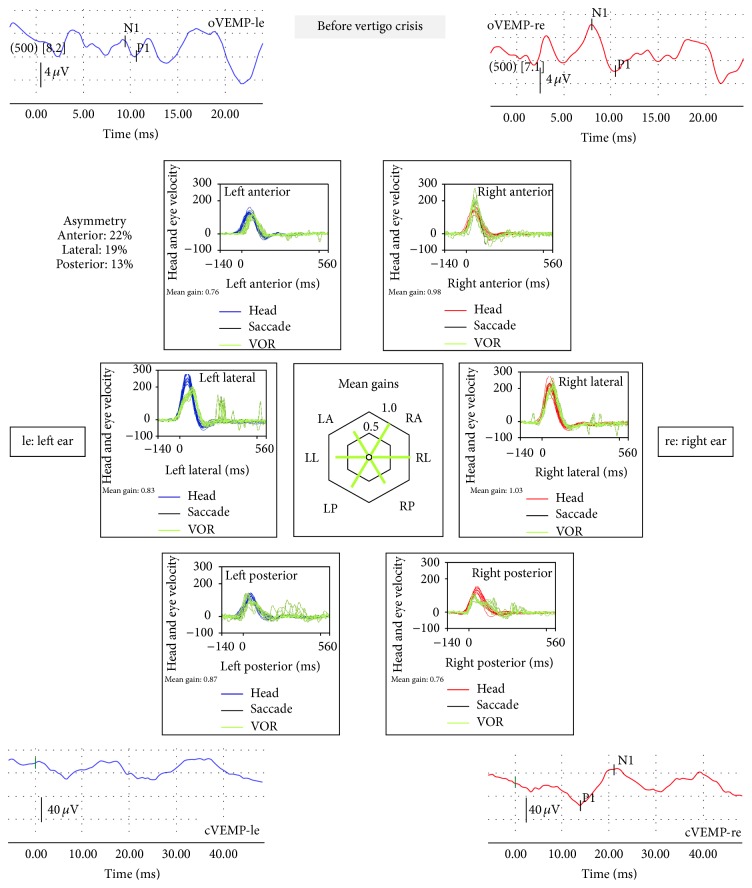
Vestibular examinations performed before the attack suffered by the patient. The vestibular myogenic evoked potentials for otolithic function, ocular (oVEMP) and cervical (cVEMP), are registered after bone vibration (500 Hz) with Bruel & Kjaer minishaker at the hairline (Fz); the response is abnormal on the left side/ear. The first component of the oVEMP (n10) is registered in the contralesional (right) eye that is very small or absent, whereas the n10 beneath the ipsilesional (left) eye is of normal amplitude. The sacculocollic cervical vestibular-evoked myogenic potential (cVEMP) is an uncrossed and predominantly sacculocollic response. The study of the vestibuloocular reflex with the video head-impulse test is normal except for the presence of reduced gain and overt saccades after left lateral semicircular canal stimulation.

**Figure 2:**
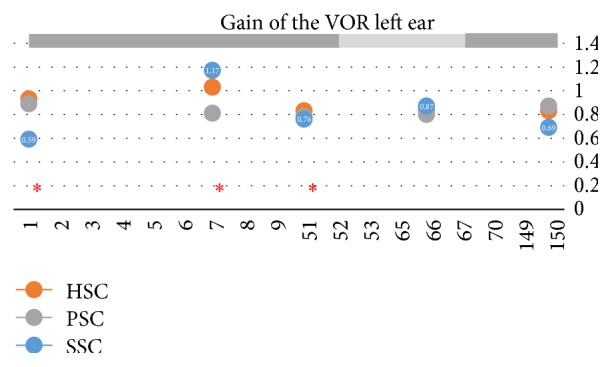
Gain of the vestibuloocular reflex in the left ear three semicircular canals at different follow-ups. Day 1 was the day of the first treatment with gentamicin intratympanically (that and the others are represented by *∗*). In light grey, the dates the patient felt steady and free of vertigo spells.

The date seen she was in good condition; last vertigo spell was 2 days before. Tympanic membrane had healed normally. There was no spontaneous nystagmus with or without visual fixation; neither gaze evoked nor post head-shake. The vHIT was normal in terms of gain of the vestibuloocular reflex (VOR), but overt saccades were clear for yaw-axis leftward head impulses as shown in Figure 1, suggesting a small reduction of VOR gain (changes in VOR at the different follows-up regarding the treatments are shown in Figure 2). Vestibular evoked myogenic potentials (VEMP) were performed with Fz 500 Hz vibration delivered with a Bruel & Kjaer minishaker. Results are shown to be normal for the right ear (both oVEMP and cVEMP) and abnormal for the left (both oVEMP and cVEMP) as shown in Figure 1. Hearing loss was mild to severe in her left ear (PTA was 48 dBHL).

**Figure 3:**
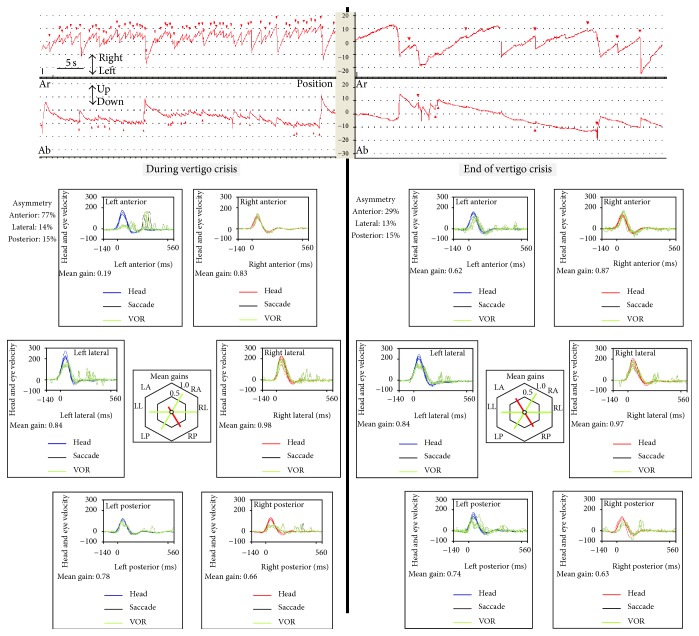
Spontaneous nystagmus and results of the video head-impulse test at the initiation of the crisis and at the end. Nystagmus is left beating with an upward component and there is a clear decrease in the gain of the vestibuloocular reflex in the plane of the left superior semicircular canal and right posterior canal (LARP) and the consequent presence of saccades. After the acute attack the same evaluation shows an increase of the gain and a significant decrease of the nystagmus.

After clinical and laboratory evaluation and when the patient was entering the office, she began to develop a vertigo spell and concurrently an intense tinnitus and pressure sensation in her left ear. At that time a left beating nystagmus with a mean slow phase velocity (mSPV) of 9.7°/s, and suppressed by visual fixation was seen under video-Frenzel glasses; 3 minutes latter a mild (mSPV = 4.3°/s) upbeat component was added (Figure 2). The nystagmus increased in leftward gaze and there was not skew deviation. In the vHIT there was an abnormal left superior VOR and this (left-anterior right-posterior) LARP plane of testing was repeated 3 times, in a period of 10 minutes, and was similar in all of them. The vertigo spell did not stop for 20 minutes and at that time was treated with sulpiride (100 mg I.M.). Three hours later the vertigo spell ended and the patient was able to return home; nystagmus was significantly reduced and vHIT returned to almost normal (precrisis) level (Figure 3).

## 3. Discussion

The situation that we present here is indicative of a complete, temporary dysfunction in the superior semicircular canal in the affected side and a unidirectional nystagmus beating ipsilaterally and upwards; in other words both horizontal and vertical components were present. These previously unreported findings while being in an acute vertigo spell in a patient with Meniere's disease deserve an explanation:

(1) While being in the vertigo crisis an acute deficit in the left superior canal was registered and confirmed not to be an artifact: the low gain was followed in each impulse by clear refixation saccades which had not been present during the earlier test in the quiescent phase.

(2) As the patient was seen in the very first minute of the crisis we can say that, in terms of nystagmus, this was unidirectional throughout the episode with a vertical component soon added and kept constant. In the case of unidirectional nystagmus most are paretic and very rarely irritative [3]. However the nystagmus in MD can change directions and the order of appearance heterogeneous. In general, an initial and brief period (<2 min) of irritative nystagmus is followed by a more prolonged period (20–30 min) during which nystagmus changes to paretic, being followed by a more prolonged period (days) of irritative nystagmus also called “recovery nystagmus.” Different sequences can occur regarding the period of time the patient is seen. The origin for nystagmus has been claimed to be due to the irritative and paralytic action of potassium in the perilymph, respectively, for the first and second periods and to be due to a fast adapting response for the third one [5, 6]. Mechanical effects have been hypothesized to explain these findings too [7].

(3) Interestingly the spontaneous nystagmus has little effect on the assessment of both horizontal semicircular canals, either at the beginning or at the end of the crisis, probably because the SPV was so small relative to SPV of the VOR response.

(4) Regarding the treatment performed we can speculate that although some of the gentamicin could make its way to the inner ear the amount must be very small according to changes in the VOR in the different periods of time the patient was seen: the function from the horizontal and posterior semicircular canals in the left ear almost did not change, while that of the superior as mentioned above showed some fluctuations. This is also supported in the examination performed before the crisis: there were signs neither of the expected acute status nor of the compensated status after an appropriate damage to the left ear: spontaneous right beating nystagmus, biphasic post-head-shake nystagmus (first right and after left beating nystagmus).

The strong unidirectional nystagmus first raised the question of a deficit from the right side; however no difference to previous test was seen and during the crisis the patient mentioned neither symptoms pointing to a right side auditory deficit nor to pressure or tinnitus. Contrary to this an aggravation of pressure and tinnitus was attributed to her left ear. The finding of a reduction in right side posterior canal function (RP) is thought to occur due to the loss of concurrent inhibitory function from the left superior (LA) canal for head impulses in their plane. Alternatively an increase in the activity from the left horizontal canal could account for the right beating nystagmus; however as same as with the right side, no changes were seen form before to during the crisis for the function in this canal.

The need to focus on the superior canal is mainly due to the finding during the crisis but also is supported by a previous report that mentions marked fluctuations in activity for the canal function between the quiescent and acute attack states and in particular after gentamicin treatment [8]. In our patient in the different follow-ups in which vHIT was done, a fluctuation in the activity of the left superior canal function was registered. In the case of a deficient left superior semicircular canal the expected nystagmus should be a mixed vertical-torsional that from the examiner's point of view should be upward and counterclockwise with a small rightward component on VNG. However in our case only the vertical component fits with this finding; the torsional component is very slow and the horizontal one is the contrary.

A very similar pattern of nystagmus has been described in patients after surgery for superior semicircular canal dehiscence [9]. In all patients there is an abnormal HIT for impulses in the plane of the treated semicircular canal as expected for a surgery that prevents endolymph flow through that canal and abnormal pressure effects related to dilation of the canal and in 40% of the patients this combines also with a deficit in the posterior canal too; in these patients nystagmus, in the immediate postoperative period, was beating to the operated side. In the infrequent situation of a complete deficit for all the three semicircular canals nystagmus was typically paretic, beating to the nonoperated side [10]. For the situation in which an irritative nystagmus was found an exchange of potassium between endolymph and perilymph was proposed to occur through small leaks in the membranous canal. This finding was also reported by others who also describe an acute event in a patient with Meniere-like symptoms but with superior semicircular canal dehiscence [11]. The pattern of the spontaneous nystagmus was similar as irritative both for the horizontal and torsional components. Concurrent with previous findings the authors presume an endolymphatic hydrops secondary to the action of prolapsing dura on membranous labyrinth through large canal dehiscences.

Another source of horizontal nystagmus has been shown to the utricle. Due to convergence of neural input from the otoliths onto horizontal canal neurons in the vestibular nuclei an ipsilesional nystagmus can occur in case of a deficit of the utricle [12]. In our case the previously done oVEMP both at the time of diagnosis (not shown) and at the day of last follow-up (Figure 1) is concurrent with a deficit from the ipsilateral utricle. However in the case of acute vertigo attacks in patients with Meniere's disease usually there is an enhancement of dynamic utricular function in the affected ear, contrary to what occurs in the case of Lermoyez crisis. We can speculate that this could have occurred in our patient and in particular because the modulation of nystagmus direction was very small when gaze was taken to right/left and up/down. Unfortunately her clinical situation was not good enough to proceed in more tests while being in the crisis.

As such, a combination of increased pressure in the left utricle (with concurrent amelioration of the n10 potential of the oVEMP, not found) generating a left beating nystagmus, transmitted to the superior semicircular canal with a concurrent utriculopetal displacement of the cupula (in the inhibitory direction) generating an upbeating nystagmus, could sum to generate the findings in our patient.
